# 

**Published:** 1906-05

**Authors:** 


					﻿Vol. XXI. -
MAT^ 1906.
No- «
TEXAS
MedtcaHJoHrn<.
M-
w
s
r
p
Hii
pq
wn
MB PI
it
M
r •«<
B
In
||
|o
|h
B
fe
|
in
® •
Subscription, $i.oo a Year, in Advance.
Single Copies, io Cents.
' PUK^fiHteD AT AUSTIN, TEXAS, KT
ri»^iVEjDANIEL, M. D., \
r Proprietor.
PRINTED BY TJ HE' VONJBGEOKMANN-JQNBS:CO/“
Entered at the Postoffice at Austin, Texas, as Second-class Mail Matter.
				

